# TT genotype of rs2941484 in the human HNF4G gene is associated with hyperuricemia in Chinese Han men

**DOI:** 10.18632/oncotarget.15851

**Published:** 2017-03-02

**Authors:** Bang-Dang Chen, Xiao-Cui Chen, Shuo Pan, Yi-Ning Yang, Chun-Hui He, Fen Liu, Xiang Ma, Min-Tao Gai, Yi-Tong Ma

**Affiliations:** ^1^ Xinjiang Key Laboratory of Cardiovascular Disease, Clinical Medical Research Institute of First Affiliated Hospital of Xinjiang Medical University, Urumqi, China; ^2^ Department of Cardiology, First Affiliated Hospital of Xinjiang Medical University, Urumqi, China; ^3^ First Department of Cardiology, People's Hospital of Shaanxi Province, Xi'an, China

**Keywords:** hyperuricemia, HNF4G, single nucleotide polymorphism, genetic, case-control association study

## Abstract

The aim of the study is to investigate the association between the human hepatocyte nuclear factor 4 gamma (*HNF4G*) gene and hyperuricemia in Chinese Han population. A total of 414 hyperuricemia patients and 406 gender and age-matched normouricemic controls were enrolled. Four single nucleotide polymorphisms were genotyped as genetic markers for the human *HNF4G* gene (rs2977939, rs1805098, rs2941484, rs4735692). Data were analyzed for two separate groups: men and women. For rs2941484, the genotype distribution frequency in hyperuricemic subjects and was significantly different from that in normouricemic controls in men (*P* = 0.038). Meanwhile, in recessive model of rs2941484, the distribution frequency of TT genotype and CC+CT genotypes also differed significantly between the hyperuricemia men and normouricemic men (*P* = 0.011). For the other 3 SNPs in both men and women, there was no difference in the genotype and allele and distribution frequency between the hyperuricemia patients and normouricemic controls. In men, after adjustments for BMI, SBP, DBP, fasting glucose, total cholesterol, triglycerides, low density lipoprotein cholesterol and creatinine, the men with the TT genotype of rs2941484 were found to have significantly higher probability of suffering from hyperuricemia than the ones with CT and CC genotypes (OR = 2.170, *P* < 0.001). Therefore, TT genotype of rs2941484 in the human *HNF4G* gene might be a gender-specific genetic marker for hyperuricemia in Chinese Han men.

## INTRODUCTION

Uric acid is a final breakdown product of purine oxidation in humans. Elevated concentrations of serum uric acid, which is named as hyperuricemia, can cause gout [1–2]. Gout is the most prevalent inflammatory arthritis in US, with an estimated 8.3 million adults having had at least one of the extremely painful attacks in 2007–2008 [3]. Via reviewing the epidemiological surveys conducted in China, the pooled prevalence of hyperuricemia and gout was 13.3% and 1.1%, respectively [4]. It is especially common in the elderly population, one quarter of Chinese population had hyperuricemia according to a recent cross-sectional study of 3978 men aged 40–74 yrs living in Shanghai, China [5].

The increasing of hyperuricemia and gout prevalence is partly owing to aging, dietary and lifestyle factors, and rising levels of obesity and insulin resistance [6–9]. The heritability of serum uric acid concentrations is estimated at 40–70% [10–12], which justifies the search for its genetic determinants. An recent genome-wide association study (GWAS) combined data from > 140,000 individuals of European ancestry within the Global Urate Genetics Consortium (GUGC) [13], they reported that single nucleotide polymorphisms (SNPs) near HNF4G was found to be associated with uric acid concentrations (*P* = 4.4 × 10–17).

Hepatocyte nuclear factor 4 gamma (HNF4G) gene in human is one of the two isoforms of human HNF4 gene, while the other isoform is hepatocyte nuclear factor 4 alpha (HNF4A). The HNF4G was originally identified in rat liver nuclear extracts as a protein binding to a DNA element of the transthyretin promoter [14]. Previous study also revealed that HNF4 is an orphan member of the nuclear receptor superfamily with a DNA binding domain and a putative ligand binding domain [15]. Binding sites for HNF4G have been found in the regulatory regions of many genes expressed in the liver and kidney [16]. Since the blood uric acid concentrations are determined by a balance between uric acid production in the liver and its disposal via the kidney and gut, we hypothesized that HNF4G may play an important role in uric acid heritability features.

Therefore, the objective of this study was to assess the genetic associations of *HNF4G* polymorphisms and hyperuricemia in Chinese Han population in Xinjiang.

## RESULTS

Table 1 shows the clinical characteristics of the study participants. The age and gender distribution frequency between the hyperuricemia patients and normouricemic controls showed no significant difference in both men and women. For both men and women, BMI, WC, SBP, total cholesterol, triglycerides, LDL-C, fasting glucose, creatinine, urea nitrogen and uric acid were significantly higher for the hyperuricemia patients as compared to the normouricemic control subjects. DBP was significantly higher for the hyperuricemia men as compared to the normouricemic control men, however, the trend was not observed in women.

**Table 1:** Characteristics of control subjects and patients with hyperuricemia

	Men		Women	
	Normouricemic controls	Hyperuricemic subjects	Normouricemic controls	Hyperuricemic subjects
Number of subjects	243	250	163	164
Age (years)	55.5 ± 8.8	55.6 ± 12.4	58.5 ± 14.8	58.4 ± 12.6
BMI (kg/m^2^)	22.2 ± 4.7	24.6 ± 4.4*	21.5 ± 3.5	23.4 ± 5.1*
WC (cm)	76.2 ± 14.2	80.5 ± 12.5*	71.3 ± 10.1	74.3 ± 12.8*
SBP (mmHg)	126.7 ± 21.1	133.3 ± 18.8*	121.3 ± 17.8	127.6 ± 24.2*
DBP (mmHg)	77.4 ± 13.9	82.9 ± 14.2*	78.21 ± 11.6	79.3 ± 14.3
Total cholesterol (mmol/L)	3.97 ± 0.86	4.11 ± 0.91*	3.95 ± 0.84	4.35 ± 1.17*
Triglycerides (mmol/L)	1.08 ± 0.23	1.33 ± 0.26*	0.94 ± 0.22	1.20 ± 0.31*
LDL-C (mmol/L)	2.21 ± 0.36	2.38 ± 0.39*	2.17 ± 0.33	2.51 ± 0.37 *
HDL-C (mmol/L)	1.13 ± 0.25	1.08 ± 0.28	1.27 ± 0.27	1.20 ± 0.27
Fasting glucose (mmol/L)	4.96 ± 0.33	5.13 ± 0.37*	4.87 ± 0.33	5.09 ± 0.37*
Creatinine (mmol/L)	80.4 ± 15.4	89.0 ± 18.6*	66.9 ± 12.8	73.0 ± 13.4*
Urea nitrogen (mmol/L)	4.57 ± 1.29	4.92 ± 1.47*	3.96 ± 1.16	4.47 ± 1.27*
Uric acid (μmol/L)	295.4 ± 63.9	460.9 ± 45.4*	237.3 ± 55.6	396.4 ± 42.1*

Note: BMI, body mass index; WC, waist circumference; SBP, systolic blood pressure; DBP, diastolic blood pressure; LDL-C, low density lipoprotein cholesterol; HDL-C, high density lipoprotein cholesterol; *P < 0.05 between the normouricemic controls and hyperuricemic subjects.

Table 2 shows the distribution frequency of the genotypes and alleles for the four SNPs. The genotype distribution frequency for each of the SNPs was in good agreement with the predicted Hardy-Weinberg equilibrium values (data not shown). For rs2941484 (SNP3), the genotype distribution frequency in hyperuricemic subjects and was significantly different from that in normouricemic controls in men (*P* = 0.038). Meanwhile, in recessive model of rs2941484, the distribution frequency of TT genotype and CC+CT genotypes also showed the significant difference between the hyperuricemia men and normouricemic men (*P* = 0.011). However, the same trend was not noticed in women. For the other 3 SNPs selected for present study, there was no difference in the genotype and allele and distribution frequency between the hyperuricemia patients and normouricemic controls.

**Table 2:** Genotyping and allele distributions in control subjects and patients with hyperuricemia

Variants	Men			Women		
	Normouricemic controls	Hyperuricemic subjects	*P* value	Normouricemic controls	Hyperuricemic subjects	*P* value
rs2977939 (SNP1)						
Genotyping						
TT	200 (82.3%)	204 (81.6%)		135 (82.8%)	142 (86.6%)	
CT	41 (16.9%)	43 (17.2%)		27 (16.6%)	22 (13.4%)	
CC	2 (0.8%)	3 (1.2%)	1.000	1 (0.6%)	0 (0%)	0.397
Dominant model						
TT	200 (82.3%)	204 (81.6%)		135 (82.8%)	142 (86.6%)	
CC+CT	43 (17.7%)	46 (18.4%)	0.839	28 (17.2%)	22 (13.4%)	0.344
Recessive model						
CC	2 (0.8%)	3 (1.2%)		1 (0.6%)	0 (0%)	
TT+CT	241 (99.2%)	247 (98.8%)	1.000	162 (99.4%)	164 (100.0%)	0.498
Allele						
C	45 (9.3%)	49 (9.8%)		29 (8.9%)	22 (6.7%)	
T	441 (90.7%)	451 (90.2%)	0.773	297 (91.1%)	306 (93.3%)	0.297
rs1805098 (SNP2)						
Genotyping						
GG	80 (32.8%)	87 (34.8%)		54 (33.3%)	58 (35.4%)	
AG	122 (50.0%)	125 (50.0%)		83 (51.2%)	87 (53.0%)	
AA	42 (17.2%)	38 (15.2%)	0.796	25 (15.4%)	19 (11.6%)	0.594
Dominant model						
AG	122 (50.0%)	125 (50.0%)		83 (51.2%)	87 (53.0%)	
AA+GG	122 (50.0%)	125 (50.0%)	1.000	79 (48.8%)	77 (47.0%)	0.743
Recessive model						
AA	42 (17.2%)	38 (15.2%)		25 (15.4%)	19 (11.6%)	
GG+AG	202 (82.8%)	212 (84.8%)	0.544	137 (84.6%)	145 (88.4%)	0.309
Allele						
A	206 (42.2%)	201 (40.2%)		133 (41.0%)	125 (38.1%)	
G	282 (57.8%)	299 (59.8%)	0.520	191 (59.0%)	203 (61.9%)	0.443
rs2941484 (SNP3)						
Genotyping						
CC	114 (46.9%)	108 (43.2%)		77 (47.2%)	74 (45.1%)	
CT	107 (44.0%)	100 (40.0%)		72 (44.2%)	72 (43.9%)	
TT	22 (9.1%)	42 (16.8%)	0.038*	14 (8.6%)	18 (11.0%)	0.757
Dominant model						
CC	114 (46.9%)	108 (43.2%)		77 (47.2%)	74 (45.1%)	
TT+CT	129 (53.1%)	142 (56.8%)	0.407	86 (52.8%)	90 (54.9%)	0.701
Recessive model						
TT	22 (9.1%)	42 (16.8%)		14 (8.6%)	18 (11.0%)	
CC+CT	221 (90.9%)	208 (83.2%)	0.011*	149 (91.4%)	146 (89.0%)	0.468
Allele						
C	335 (68.9%)	316 (63.2%)		226 (69.3%)	220 (67.1%)	
T	151 (31.1%)	184 (36.8%)	0.058	100 (30.7%)	108 (32.9%)	0.536
rs4735692 (SNP4)						
Genotyping						
GG	94 (38.7%)	93 (37.2%)		60 (36.8%)	66 (40.2%)	
AG	108 (44.4%)	114 (45.6%)		72 (44.2%)	73 (44.5%)	
AA	41 (16.9%)	43 (17.2%)	0.944	31 (19.0%)	25 (15.2%)	0.627
Dominant model						
AG	108 (44.4%)	114 (45.6%)		72 (44.2%)	73 (44.5%)	
GG+AA	135 (55.6%)	136 (54.4%)	0.797	91 (55.8%)	91 (55.5%)	0.951
Recessive model						
AA	41 (16.9%)	43 (17.2%)		31 (19.0%)	25 (15.2%)	
GG+AG	202 (83.1%)	207 (82.8%)	0.923	132 (81.0%)	139 (84.8%)	0.365
Allele						
G	296 (60.9%)	300 (60.0%)		192 (58.9%)	205 (62.5%)	
A	190 (39.1%)	200 (40.0%)	0.771	134 (41.1%)	123 (37.5%)	0.345

Note: *P < 0.05 between the normouricemic controls and hyperuricemic subjects.

Since the TT genotype distribution frequency of rs2941484 was significantly higher in hyperuricemic men as compared to the normouricemic control men, we may identify TT genotype as a risk factor for hyperuricemia in Chinese Han men. Therefore, the logistic regression analysis was performed using TT genotype of rs2941484 and each of the risk factors associated with the hyperuricemia (Table 3). In men, after adjustments for BMI, SBP, DBP, fasting glucose, total cholesterol, triglycerides, LDL-C and creatinine, the men with the TT genotype of rs2941484 were found to have significantly higher probability of suffering from hyperuricemia than the ones with CT and CC genotypes (*OR* = 2.170, *P* < 0.001). Meanwhile, we also found BMI, fasting glucose, total cholesterol, LDL-C, creatinine were associated with hyperuricemia in Chinese Han men (all *P* < 0.05).

**Table 3:** Logistic regression analysis of confounding factors associated with hyperuricemia

Risk factors	Odd ratios	95% CI	*P* value
TT genotype of rs2941484	2.170	1.348–2.992	< 0.001*
BMI	2.912	1.988–3.837	< 0.001*
SBP	1.149	0.867–1.431	0.412
DBP	1.091	0.753–1.429	0.766
Fasting glucose	2.798	1.983–3.613	< 0.001*
Total cholesterol	1.531	1.101–1.961	0.029*
Triglycerides	1.133	0.780–1.636	0.122
LDL–C	1.287	1.004–1.570	0.047*
Creatinine	2.106	1.762–2.451	< 0.001*

Note: BMI, body mass index; SBP, systolic blood pressure; DBP, diastolic blood pressure; LDL–C, low density lipoprotein cholesterol; *P < 0.05 between the normouricemic controls and hyperuricemic subjects.

## DISCUSSION

Accumulating evidence suggests that hyperuricemia is one of the important factors that may significantly contribute to the development and progression of the cardiorenal and metabolic disorders [17, 18]. Elevated levels of uric acid were found to be associated with inflammation, oxidative stress, insulin resistance, dysglycemia, endothelial dysfunction, vascular, renal and cardiac stiffness, cardiac diastolic dysfunction, renal hyperfiltration and proteinuria [19–21]. Studies of twins and families have shown the inheritance for both hyperuricaemia and excretion of urate via the kidneys [22]. In one study in twins, the heritability of the renal clearance of urate was 60% to 87% [23]. Other studies have shown that serum urate levels have substantial heritable traits (40%) [24]. Over the past decade, GWAS, replication studies and meta-analyses have led to a remarkable increase in our knowledge of common genetic variants that predispose to hyperuricaemia and gout. An recent genome-wide association studies (GWAS) combined data from > 140,000 individuals of European ancestry within the Global Urate Genetics Consortium (GUGC) [13], they reported 18 new loci associated with serum urate concentrations. Among them, rs2941484 of *HNF4G* was found to be associated with uric acid concentrations with the effect value was 0.044 (*P* = 4.4 × 10–17). However, the OR value for the gout is 1.04, the difference was not significant ( *P* = 1.7 × 10−1).

HNF4 was first identified as a DNA binding activity in rat liver nuclear extracts. With protein purification technique, the cDNA of rat HNF4 was cloned and HNF4 was found to be an orphan member of the nuclear receptor superfamily [14, 26]. Binding sites for HNF4 were identified in many tissues, the expressed genes and the proteins were found to be essential for early embryonic development in the mouse [26]. *HNF4G* gene in human is one of the two isoforms of human *HNF4* gene, while the other isoform is *HNF4A*. HNF4G is a member of the HNF4 orphan subfamily [27]. It can be expressed in the pancreas, kidney, small intestine and testis, however, it can not be expressed in the human liver [14]. The gene regulation effected by *HNF4G* has been reported to occur in coordination with *HNF4A* [28–30].

In this present study, the genotype frequency of rs2941484 showed significantly difference between the hyperuricemia men and normouricemic men, the men with the TT genotype of rs2941484 were found to have significantly higher probability of suffering from hyperuricemia than the ones with CT and CC genotypes after adjustments for BMI, SBP, DBP, fasting glucose, total cholesterol, triglycerides, LDL-C and creatinine. The results of our study was consistent with the GWAS on serum urate concentrations [13], we found that the TT genotype of rs2941484 in *HNF4G* gene was associated with hyperuricemia only in Chinese male population. Some case-control studies have previously identified gene variants that are associated with gender-specific susceptibility to hyperuricemia [31, 32], they both reported the gender-specific gene for hyperuricemia and gout in men rather than in women [33, 34]. The sex specificity demonstrated in the present study was not clear yet. The possible reasons may be as follows: the dominant number of male hyperuricemia and gout patients over female ones, meanwhile, the female estrogen can promote the excretion of uric acid and inhibit the onset of arthritis. The two reasons above may make a large sample of female hyperuricemia patients difficult to collect, then the probability of discovering new genetic variants might be low. Therefore, the association between the genetic variants and hyperuricemia in men was much easier to be discovered.

The mechanism by which *HNF4G* may be associated with hyperuricemia is still unclear. Many researchers have discovered the association of *HNF4G* with obesity, since rising levels of obesity can contribute greatly to increasing prevalence of hyperuricemia and gout. We hypothesized that the association of *HNF4G* with hyperuricemia may be established via its association with obesity. In this paper, we have also confirmed that BMI is significant risk factor for hyperuricemia in the logistic regression (*OR* = 2.912, *P* < 0.001). Meanwhile, in a GWAS study including up to 263,407 European individuals [35], an SNP near *HNF4G* (rs4735692) was found to be associated with clinical classes of obesity. A gene knockout mice experiment found that the *HNF4G* knockout (*HNF4 gamma* (−/−)) mice had lowered energy expenditure and locomotor activity during night time that resulted in a higher body weight when compared with littermate wild-type mice (*HNF4 gamma* (+/+)) [36]. The increased probability of obesity caused by lowered energy expenditure and locomotor activity may subsequently increase the probability of hyperuricemia. In one large animal experiment, Ramayo-Caldas et al [37] found that HNF4G had the regulatory role in intramuscular fat deposition of beef cattle. Meanwhile, the intramuscular fat percentage and body adiposity have been proved to be the good predictors for the obesity status [38]. At the molecular level, one study suggested that the HNF4G may be transcription factors that are constitutively bound to fatty acids [39]. Since the plasma free fatty acid may be the reasons leading to obesity and insulin resistance, *HNF4G* may be associated with hyperuricemia from its association with fatty acids levels.

The present study has the significant strength. First, the SNPs near *HNF4G* was found to be associated with uric acid concentrations by GWAS based on large sample of European decedents, the confirmation of the results in Chinese Han population for the first time may contributed greatly to the in-depth studies of the gene. Second, all the hyperuricemia subjects and gender and age-matched normouricemic controls were selected based on the Cardiovascular Risk Survey (CRS) study, which is the representative sample of the general adult Han population in China. Thus, these genotyping results can be generalized to the full adult Han population aged above 35 years in China.

The limitation of this study is based on its cross-sectional sample population, it can't reflect the causal relationship between the *HNF4G* gene and hyperuricemia. Second, we did not include ethnic specified data in the paper since the prevalence of hyperuricemia in Uygur and Kazakh population was too low for a genetic association study. Third, the since the female hyperuricemia patients were difficult to collect, the detection power of discovering the new genetic variants may decrease in women.

In conclusion, this is first time that association between the human *HNF4G* gene and hyperuricemia has been examined in the Chinese Han population. The present data indicates that TT genotype of rs2941484 in the human *HNF4G* gene might be a gender-specific genetic marker for hyperuricemia in Chinese Han men.

## MATERIALS AND METHODS

### Ethical approval of the study protocol

Written informed consent was obtained from all participants. All participants explicitly provided permission for DNA analysis as well as collection of relevant clinical data. This study was approved by the Ethics Committee of the First Affiliated Hospital of Xinjiang Medical University (Urumqi, China). It was conducted according to the standards of the Declaration of Helsinki.

### Subjects

All the hyperuricemia subjects and gender and age-matched normouricemic controls were selected from the Cardiovascular Risk Survey (CRS) study, the detailed description of the study population and the methods were described previously [40–42]. Briefly, the CRS study used a 4-stage stratified sampling method to select a representative sample of the general population in Xinjiang, northwest of China. The research sites included Urumqi City, Kelamayi City, Fukang City, Turpan Prefecture, Hetian Prefecture, Yili Prefecture. The time period was from October 2007 to March 2010. The selections made from sampling units were based on geographic area, sex, and age groups using household registries. In total, the CRS included 14 618 participants (5757 Hans, 4767 Uygurs, and 4094 Kazakhs). In this present study, we only selected our subjects based on the Chinese Han population in Xinjiang.

In this study, hyperuricemia was defined as SUA ≥ 7 mg/dl (416 mmol/L, male) or SUA ≥ 6 mg/dl (357 mmol/L, female) [43–45]. The patients may have hypertension (The diagnosis of hypertension was established if patients were on antihypertensive medication or if the mean of 3 measurements of systolic blood pressure (SBP) ≥140 mmHg or diastolic blood pressure (DBP) ≥ 90 mmHg, respectively), or diabetes mellitus (The diagnosis of diabetes mellitus was established if fasting plasma glucose ≥ 7.0 mmol/L, or with a history or treatment of diabetes). In addition, individuals with history of lowering serum uric acid agents were excluded. All enrolled subjects should have the serum blood urea nitrogen, serum creatinine within the normal range, the normal range of serum blood urea nitrogen was 3.2-7.1 mmol/L in both men and women, while the normal range of serum creatinine was 53-106 umol/L for men and 44-97 umol/L for women, respectively.

In total, we enrolled a total of 414 Han (250 male and 164 female) hyperuricemia patients. 406 Han (243 male and 163 female) gender and age-matched normouricemic subjects were enrolled as the controls. The age of enrolled patients ranged from 35 to 88. The average uric acid was 379.32 ± 54.52 μmol/L for men and 317.10 ± 48.83 μmol/L for women.

### SNP selection

*HNF4G* gene in human is one of the two isoforms of human *HNF4* gene (the other isoform being *HNF4A*). It consists of 408 amino acids and is located on chromosome 8q21.11. This gene consists approximately 26.88 kilobase pairs (kbp) and contains ten exons, which are separated by nine introns.

**Figure 1:**
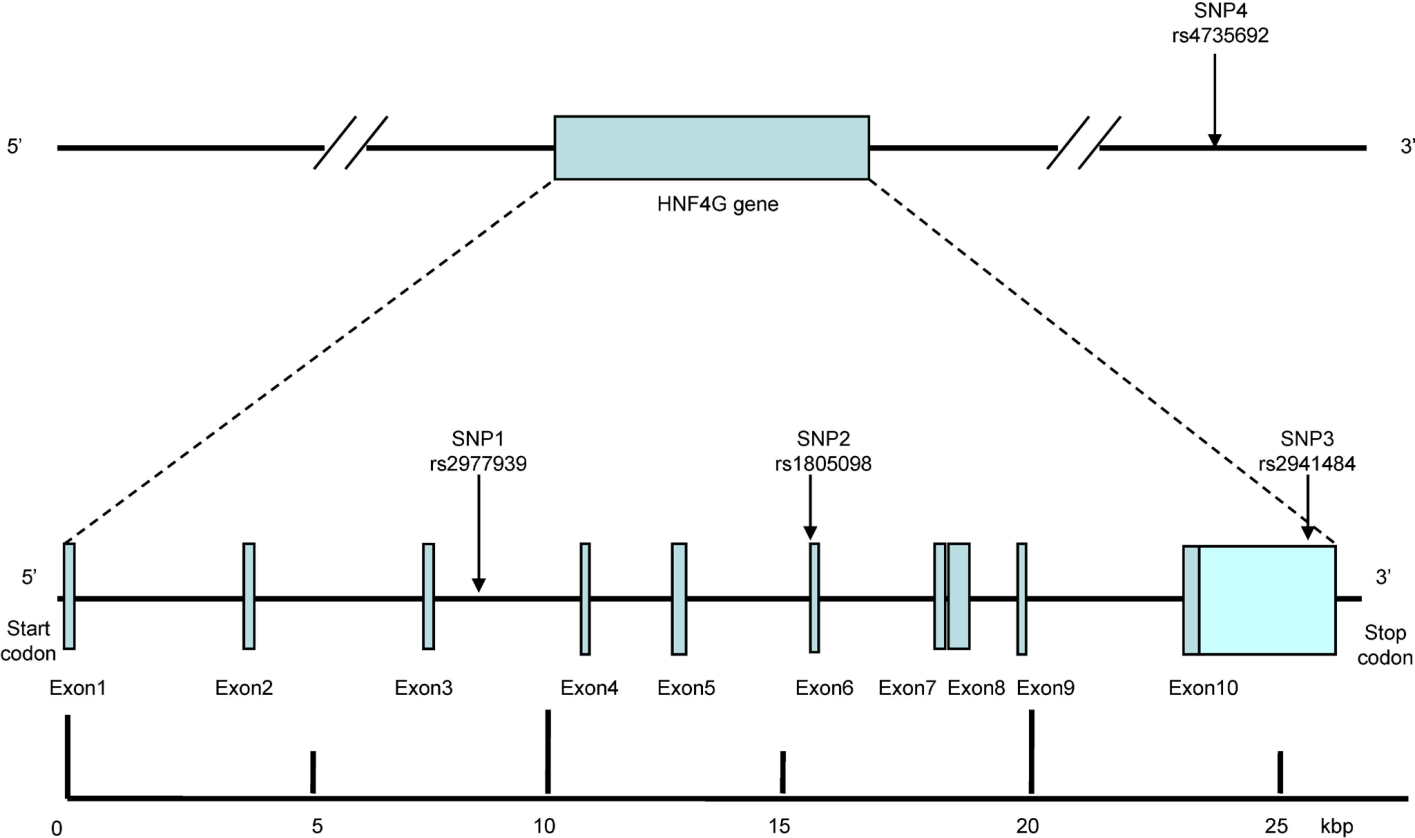
Structure of the human *HNF4G* gene The gene consists of ten exons (boxes) separated by nine introns (lines; intergenic regions). Filled boxes indicate the coding regions, while arrows indicate the locations of single-nucleotide polymorphisms (SNPs). SNP1 is located in the introns area between the exon 3 and exon 4. SNP2 is located in the exon 6. SNP3 is located in the stop codon. SNP4 is located in downstream area of *HNF4G* gene. kbp, kilobase pairs.

There are 1354 SNPs of the human *HNF4G* gene listed in the National Center for Biotechnology Information SNP database (http://www.ncbi.nlm.nih.gov/SNP). In this study, we screened the data on the International HapMap Project website (http://hapmap.ncbi.nlm.nih.gov/index.html.en) for the Tag SNPs of *HNF4G* gene. SNPs with relatively high minor allele frequencies (MAF) have been shown to be useful as genetic markers in genetic association studies. In this condition, there were four SNPs (rs2977939, rs1805098, rs2941484, rs2941465), which had a MAF of > 0.1 and r2 cutoff of 0.5 among Chinese Han in Beijing as the markers. All the four SNPs are located within or near the *HNF4G* gene (Figure 1). However, we failed to find the the TaqMan SNP Genotyping Assays for rs2941465 at the Applied Biosystems (ABI) website (http://www.lifetechnologies.com/order/genome-database). We excluded rs2941465, which was tagged only by itself from our experiment. Meanwhile, rs4735692, which was found to be associated with clinical classes of obesity in GWAS were also included in the experiment [35]. (Figure 1)

We designated the four SNPs as SNP1 (rs2977939, C_26362437_10), SNP2 (rs1805098, C_2158198_20), SNP3 (rs2941484, C_15867636_10) and SNP4 (rs4735692, C_31080394_10), which were in order of increasing distance from the 5′ end of the gene (Figure 1).

### DNA extraction and laboratory methods

Blood samples were obtained from an antecubital vein into EDTA tubes in the morning after an overnight fasting period. All the collected samples were transported on dry ice at prearranged intervals to Xinjiang Key Laboratory of Cardiovascular Disease Research. The genomic DNA was extracted from the peripheral blood leukocytes using phenol and chloroform extraction method [46]. The serum concentration of serum total cholesterol, triglyceride, low density lipoprotein (LDL), high density lipoprotein (HDL) and fasting glucose were measured by the Clinical Laboratory Department of the First Affiliated Hospital of Xinjiang Medical University with the biochemical analyzer (Dimension AR/AV Clinical Chemistry System, Newark, NJ, USA) [47].

### Genotyping

Genotyping was performed using the TaqMan SNP Genotyping Assay (Applied Biosystems). The primers and probes used in the TaqMan SNP Genotyping Assays (Applied Biosystems) were chosen based on information available at the ABI website (http://www.lifetechnologies.com/order/genome-database).

PCR amplification was performed using 2.5 μl of TaqMan Universal Master Mix, No AmpErase UNG (2×) (Applied Biosystems) in a 5 μl final reaction volume, along with 2 ng DNA, 2.375 μl ultrapure water, 0.079 μl Tris-EDTA (TE) buffer (1×), 0.046 μl TaqMan SNP Genotyping Assay Mix (40×) containing a 331.2 nmol/l final concentration of primers and a 73.6 nmol/l final concentration of the probes. The thermal cycling conditions were as follows: 50°C for 2 min; 95°C for 10 min; 50 cycles of 95°C for 15 s; and 60°C for 1 min [48].

Each 96-well plate contained 80 DNA samples of an unknown genotype and four reaction mixtures containing reagents but no DNA (control). The control samples without DNA are a necessary part of the sequence detection system of 7900 signal processing system, as outlined in the TaqMan Allelic Discrimination Guide (Applied Biosystems). The plates were read on the sequence detection system 7900 instrument with the end-point analysis mode of the sequence detection system version 1.6.3 software package (Applied Biosystems). The genotypes were determined visually based on the dyecomponent fluorescent emission data depicted in the X-Y scatter-plot of the sequence detection system software. The genotypes were also determined automatically by the signal processing algorithms of the software. The results of each scoring method were saved in two separate output files for later comparison.

### Statistical analysis

Statistical analyses were performed using SPSS software for Windows, version 17.0 (SPSS, Chicago, IL). All continuous variables were expressed as mean ± s.d. Differences in continuous variables between the hyperuricemia patients and normouricemic control subjects were analyzed using Mann-Whitney *U*-test. Differences in categorical variables between the hyperuricemia patients and normouricemic control subjects were analyzed using the chi square or Fisher's exact test. Differences in distributions of genotypes and alleles were analyzed using Fisher's exact test. Statistical significance was established at *P* < 0.05.
